# Cardiac Tamponade by Hydatid Pericardial Cyst: A Rare Case Report

**DOI:** 10.5812/aapm.9137

**Published:** 2013-12-18

**Authors:** Anil Kumar Paswan, Shashi Prakash, Rajeev K Dubey

**Affiliations:** 1 Department of Anesthesia, Institute of Medical Science, Banaras Hindu University, Varanasi, India

**Keywords:** Pericardium, Echinococcosis, Heart Failure, Angina Pectoris

## Abstract

**Introduction:**

Hydatid cysts are most commonly found in the liver and lungs but they are rarely found in pericardium.

**Case Presentation:**

We present a rare case of isolated hydatid cyst in pericardium of heart of a 70 year old female presented in casualty with unusual features like, dyspnea, palpitation and chest pain mimicking acute coronary syndrome.

**Discussion:**

Hydatid cyst in Pericardium represents only 0.5-2% of cases of systemic echinoccocal infection. Isolated pericardial cyst is very rare in endemic region and may present mimicking acute coronary syndrome. Cardiac hydatid cysts should always be considered in presence of eosinophilia as present like acute coronary syndrome in endemic area.

## 1. Introduction

Hydatid cyst is an important parasitic infection in various sheep- and cattle-raising areas in the world. In human beings it can be found in any organ or tissue but it’s rarely found in pericardium. Within the heart usually situated in the left or right ventricle (1) and rarely found in the pericardium and comprises of 0.5% to 2% of all hydatid cyst cases (2). It does not become symptomatic and this condition is often painless and silent, until the cysts grow to a large size. Complications have been reported, such as cyst rupture, cardiac compression, atrial fibrillation, and even sudden death (3). We describe the case of a patient who had pericardial hydatid cyst compression of the left ventricle and who clinically presented like acute coronary syndrome with symptoms of dyspnea, palpitation and chest pain. This is an area of endemic zone of hydatid cyst and we were operated multiple number of case of liver and lung hydatid cyst but pericardial hydatid cyst operated first time in my ten years of clinical experience.

## 2. Case Presentation

**Figure 1.**
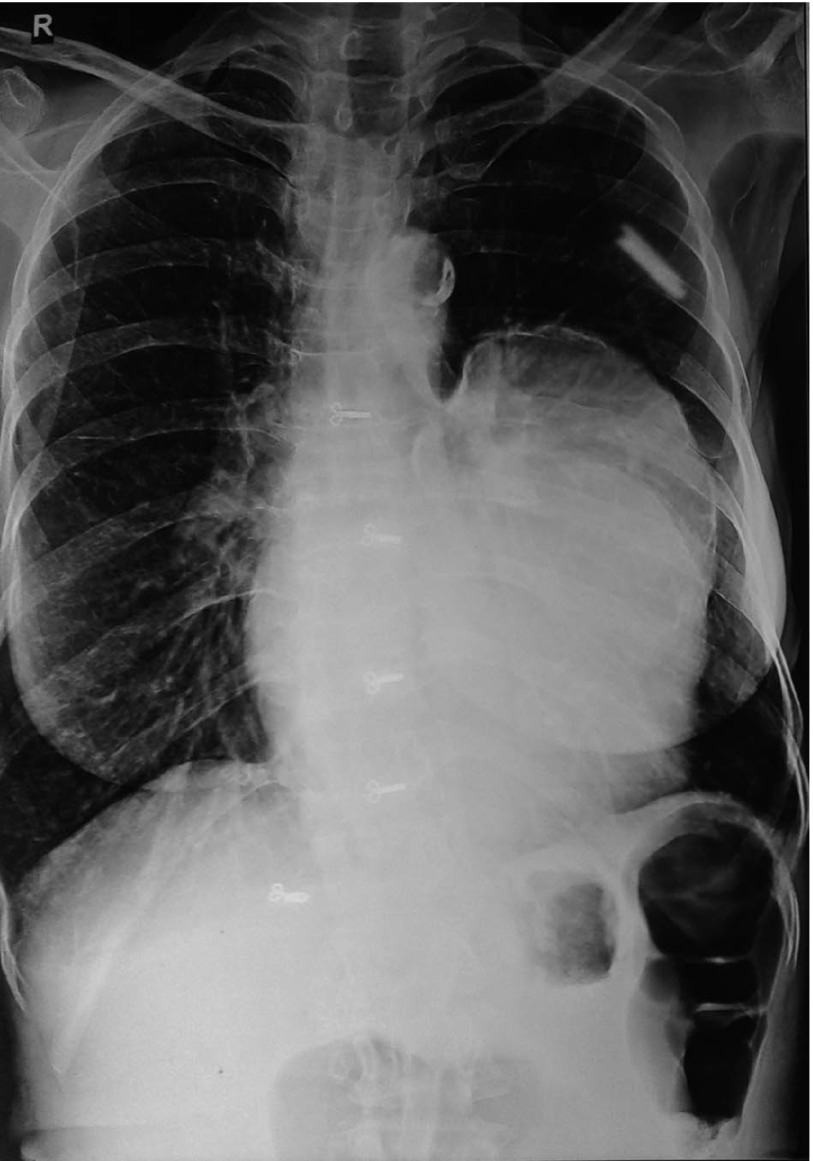
Chest X-Ray Showing Bulging of Left Heart Border

**Figure 2.**
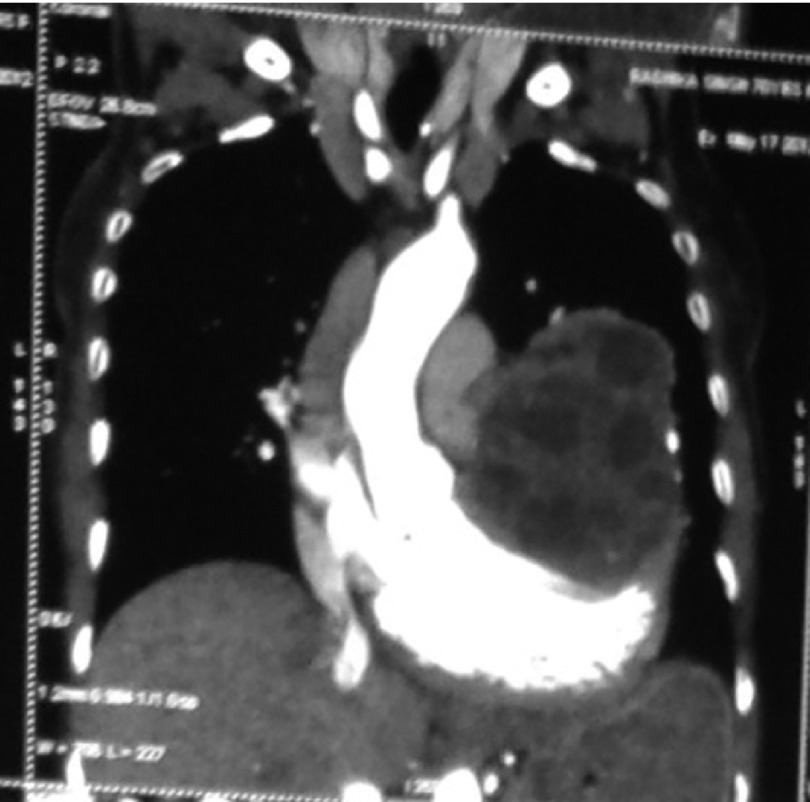
CT Scan Showing Hydatid Cyst in Pericardium

**Figure 3.**
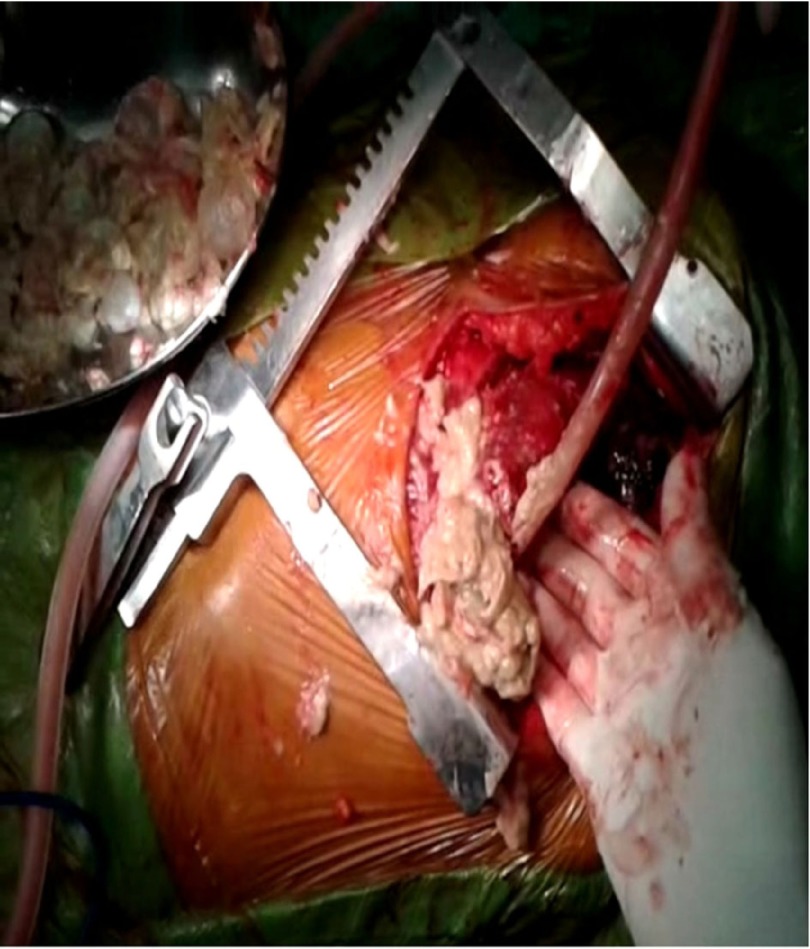
Pericardial Cyst Like Cheese Mixed With Grape Removing From Pericardium

In June 2011, a 60 year old woman was admitted to our emergency room with complaints of shortness of breath, palpitation, chest pain and progressive dyspnea. She had history of dyspnoea on exertion and orthopnea too. She had no history of trauma or major illness. On her physical examination, arterial blood pressure was 135/90 mmHg, heart rate was 110 beats per minute, SpO_2 _94% on room air and respiratory rate was 24 breaths per minute. On auscultation, the lungs were normal and no cardiac murmur, no JVP raised or gallop rhythm was noted. Cardiac enzymes, biochemical analysis, and complete blood count were normal except for eosinophilia. Chest radiography noted that cardiomegaly with bulging left heart boarder at admission (Figure 1). Electrocardiography (ECG) showed normal sinus rhythm with poor progression of R wave in lead v4, v5, and v6 and no changes in ST segment. Her myocardial-specific enzyme values were found in the normal range. Transthoracic echocardiographic investigation showed a large multicystic mass (size 77 × 57 mm) seen intrapericardially extending from left ventricle lateral wall to pericardial sac causing pressure effect on left ventricle. The abdominal ultrasonography revealed intracardiac hydatid and other cystic lesion either in the liver or other abdominal organs were not found. Contrast-enhanced CT confirmed the presence of a well-defined, thin-walled, homogeneous multiple cardiac hydatid cyst with internal trabeculae arising from pericardium with adjacent structure (Figure 2). Results of serologic tests for hydatidosis (indirect hemagglutination tests) were positive for E. granulosus, and marked eosinophilia was present too. After diagnosis, the patient was scheduled for surgery with the diagnosis of cardiac hydatid cyst. Patient was taken into the operation theatre and monitoring including ECG, pulse oximetry and non-invasive blood pressure were established and general anesthesia was performed with opioid-muscle relaxant technique after that established left radial artery cannulation for beat to beat blood pressure monitoring and internal juglar vein cannulation for central venous pressure monitoring. The pericardium was accessed through left lateral thoracotomy, accessing to the left mediastinal area, a solitary cystic lesion with a well-defined border was visualized in the pericardium adjacent to the left ventricle and the pericardial cyst was surgically removed (Figure 3). Patient’s trachea extubated and Intraoperative and postoperative hemodynamic status was maintained and within normal limits. Post-operative pain managed with inter costal block with 0.5% of bupivacain plain, inj. tramadol and diclofenac sodium I.M. Pathologic examination of the specimen was shown with hydatid cyst.

## 3. Discussion

Although hydatid disease is a parasitic infection that is endemic in many parts of the world and pericardial hydatid cysts are very unusual and rare disease as we experienced and as is evident in literature. The anatomical site of location in the heart depends on the amount of regional vascularization. Salati et al. found that in 18 year period, isolated cysts in pericardium is only 11 (1.4%) of 783 cases of cardiothoracic hydatid cysts (4) Polat P et al. reported that 50%-60% of the cardiac hydatid cysts are located in the left ventricle, 10%-20% are located in the interventricular septum, 5%-15% are located in the right ventricle, 10%-15% are located in the pericardium, and 5%-8% are located in the right or left atrium (5). Akar et al. found that in 60 patients of case series, left ventricle is the most common site of location by 47%, followed by the right ventricle (19%), interventricular septum (19%) and right atrium (11%) (6). Isolated hydatid cyst in pericardium reported as a rare entity (7). Alae F et al. (8) has shown that thoracic computerized tomography and magnetic resonance imaging showed a large cyst measuring 5 × 3 cm in the interventricular septum, a smaller cyst in the left ventricular lateral wall and a cyst at the upper lobe of the left lung . Geramizadeh B et al. (9, 10) has reported that three case of alveolar echinococcus of liver and Isolated Adrenal gland hydatid cyst in different geographic region of Iran. In the present case, the hydatid cyst was located in the pericardium adjacent to the left ventricle. Symptoms of a pericardial hydatid cyst are generally due to the pressure exerted on the myocardium by an enlarging cyst or due to rupture of the cyst. Because the pericardium is non-compliant, increases intrapericardial pressure resultant adverse cardiac compressive effect on cardiac filling and output and clinical manifestations of cardiac tamponade, constrictive pericarditis may arose. Patients with cardiac hydatid cysts symptoms can be quite variable, most commonly present to the emergency department with dyspnea, palpitation, and chest pain mimicking coronary syndromes (7) as in our case. However, echocardiography and MRI/ CT remain the best techniques for diagnosing and locating cardiac hydatid cysts. According to our clinical experiences, the frequency of hydatid cysts by organs in order of decreasing in frequency is following: liver, pulmonary, and cardiac. This area is an endemic zone of hydatid cyst and we were operated multiple number of case of liver and lung hydatid cyst but isolated pericardial hydatid cyst operated first time in my ten years of clinical experience. In a patient with a history of hydatid disease in endemic region, the rupture of a hydatid cyst should be considered as a possible diagnosis when circulatory collapse is the initial symptom. This case is reported because of the diagnostic problems encountered mimicked acute coronary syndrome and it is rare disease even in endemic zone like our state Eastern UP of India. Cardiac hydatid cysts should always be considered in presence of eosinophilia in the differential diagnosis of pericarditis or pericardial effusion, especially in regions where hydatid disease is endemic.
